# Deciphering alternative splicing and nonsense-mediated decay modulate expression in primary lymphoid tissues of birds infected with avian pathogenic *E. coli* (APEC)

**DOI:** 10.1186/s12863-017-0488-4

**Published:** 2017-03-07

**Authors:** Hongyan Sun

**Affiliations:** College of Animal Science and Technology, Yangzhou University, Yangzhou, Jiangsu 225009 China

**Keywords:** Alternative splicing, Nonsense-mediated decay, Bone marrow, Thymus, Bursa

## Abstract

**Background:**

Avian pathogenic *E. coli* (APEC) can lead to a loss in millions of dollars in poultry annually because of mortality and produce contamination. Studies have verified that many immune-related genes undergo changes in alternative splicing (AS), along with nonsense mediated decay (NMD), to regulate the immune system under different conditions. Therefore, the splicing profiles of primary lymphoid tissues with systemic APEC infection need to be comprehensively examined.

**Results:**

Gene expression in RNAseq data were obtained for three different immune tissues (bone marrow, thymus, and bursa) from three phenotype birds (non-challenged, resistant, and susceptible birds) at two time points. Alternative 5′ splice sites and exon skipping/inclusion were identified as the major alternative splicing events in avian primary immune organs under systemic APEC infection. In this study, we detected hundreds of differentially-expressed-transcript-containing genes (DETs) between different phenotype birds at 5 days post-infection (dpi). DETs, *PSAP* and *STT3A*, with NMD have important functions under systemic APEC infection. DETs, *CDC45*, *CDK1*, *RAG2*, *POLR1B*, *PSAP*, and *DNASE1L3*, from the same transcription start sites (TSS) indicate that cell death, cell cycle, cellular function, and maintenance were predominant in host under systemic APEC.

**Conclusions:**

With the use of RNAseq technology and bioinformatics tools, this study provides a portrait of the AS event and NMD in primary lymphoid tissues, which play critical roles in host homeostasis under systemic APEC infection. According to this study, AS plays a pivotal regulatory role in the immune response in chicken under systemic APEC infection via either NMD or alternative TSSs. This study elucidates the regulatory role of AS for the immune complex under systemic APEC infection.

**Electronic supplementary material:**

The online version of this article (doi:10.1186/s12863-017-0488-4) contains supplementary material, which is available to authorized users.

## Background

Avian pathogenic *E. coli* (APEC) can cause numerous diseases, such as colibacillosis, septicemia, pericarditis, and airsacculitis, leading to significant economic loss in the poultry industry because of mortality and produce contamination [1, 2]. Researchers have also indicated that APEC and human extraintestinal pathogenic *E. coli* (ExPEC) shared similar structures, indicating the zoonotic risk attributed to APEC strains [3–5]. Therefore, retail contaminated chicken can be considered an important reservoir for APEC to cause ExPEC infections in humans.

Studies have demonstrated that numerous immune-related genes are undergoing changes in alternative splicing (AS) to regulate the immune system under different conditions [6, 7], such as *CD3*, *CTLA4*, *CD44*, *FYN*, and *VAV1* [6–10]. In addition, non-sense mediated decay (NMD), another post-transcriptional mechanism for gene expression regulation, mainly occurs under pathogen-induced stress [11]. Kalyna et al. [12] reported on the combination of AS and NMD to regulate gene expression, suggesting that AS-NMD can control a number of splicing proteins. Lewis et al. [13] also revealed that AS-NMD is a widely used post-transcriptional regulatory strategy. Thus, studying the two major sources of transcriptome diversity - differential splicing and NMD - is critical to understanding the mechanisms of AS and its regulation among different conditions, uncovering structural and functional diversity.

Research on AS in different chicken tissues under various conditions is rarely conducted. Chacko and Ranganathan [14] indicated that about 23% of chicken genes undergo AS. Thus, the underlying post-transcriptional stage molecular mechanisms of hosts need to be elucidated to improve APEC infection response, as well as the control and prevention of the disease. In the present study, we determined the landscape of AS and NMD in the primary lymphoid tissues (bone marrow, thymus, and bursa) of birds with systemic APEC infection to offer insights into the regulatory elements that contribute to the pathobiology of APEC infection.

## Results

All libraries were sequenced by using Illumina® HiSeq 2000 with 100 bp single-end reads. RNAseq obtained approximately 21–28 million raw sequence read for each treatment for each of the three immune tissues except treatment non-challenged birds at 1 day post-infection (dpi) in bursa (Additional file 1: Figure S4). Trimmed reads that passed the quality filter were about 21–27 million (Additional file 1: Figure S4). On average, 80.52% (bone marrow), 80.93% (bursa), and 78.62% (thymus) were uniquely mapped to the chicken reference transcriptome (Additional file 1: Figure S4). Moreover, the spliced reads comprised an average of 26,16, 20.18, and 20.54% of the unique mapped reads for bone marrow, bursa, and thymus, respectively (Additional file 1: Figure S4). These results showed that more spliced reads were detected in the bone marrow than in the bursa and the thymus. Moreover, both resistant and susceptible birds had more spliced reads compared with non-challenged birds in the bone marrow and the thymus at both time post-infection (Additional file 1: Figure S4). Cufflinks was used to estimate transcript abundance with an expression-level threshold of FPKM ≥ 0.5. The sensitivity and specificity were about 90 and 45%, respectively, at the transcript level for each sample by using the Cuffcompare software.

**Fig. 1:**
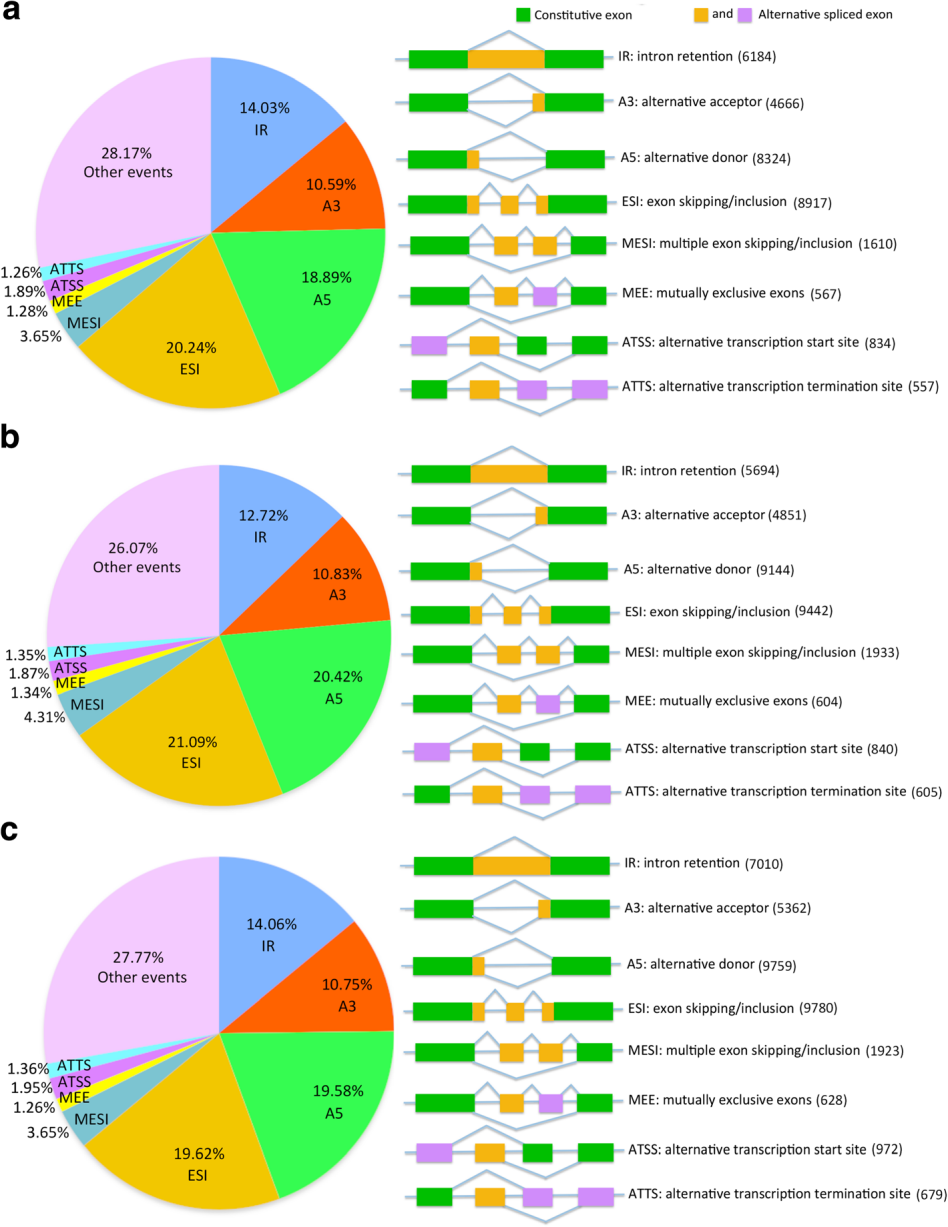
The distribution of alternative splicing events in each of chicken three primary lymphoid tissues. **a** The distribution of alternative splicing events in bone marrow. **b** The distribution of alternative splicing events in bursa. **c** The distribution of alternative splicing events in thymus

Overall, the percent of alternative splicing (AS) events was similar in each of the primary lymphoid tissues of different phenotype birds. The most common AS events were alternative 5′ splice sites (A5SS), exon skipping/inclusion (ESI), intron retention (IR), and alternative 3′ splice sites (A3SS), with 20.24, 18.89, 14.03, and 10.59%, respectively, in the bone marrow (Fig. 1a). Similar phenomenon was also detected in the bursa and the thymus (Fig. 1b and c). Collectively, the A5SS and ESI were the major AS events for chicken primary immune organs under systemic APEC infection.

Nine 2-way contrasts were generated among the six experimental groups in each of the three tissues (Additional file 1: Figure S1-3). However, only the 5 dpi contrasts included large numbers of differentially expressed (DE) isoforms in genes in all the three tissues (Additional file 1: Figure S1-3): susceptible vs. non-challenged birds at 5 dpi and susceptible vs. resistant birds at 5 dpi.

**Fig. 2:**
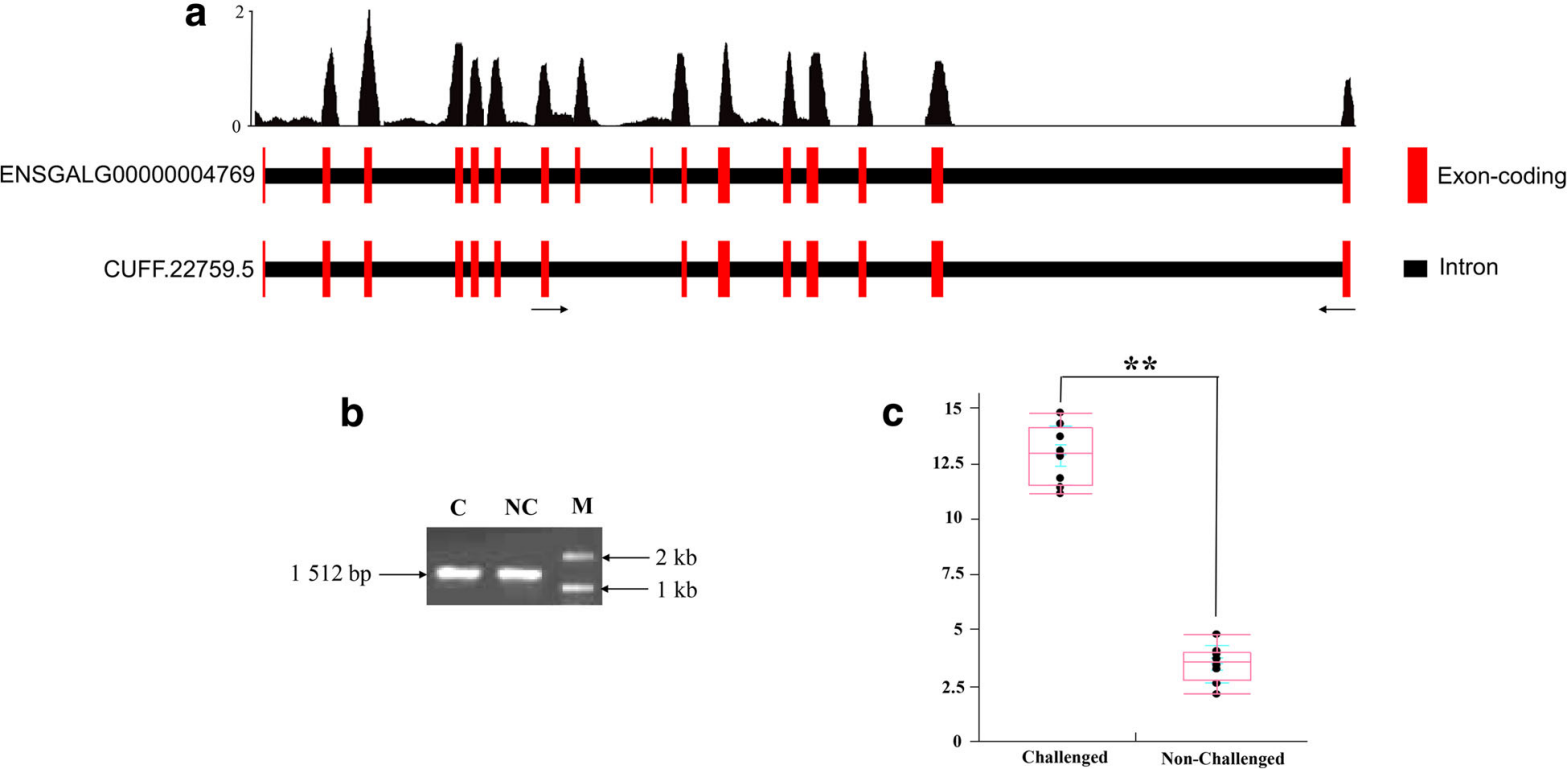
Expression level of *PSAP* transcript CUFF.22759.5 in the thymus of challenged and non-challenged birds by RT-qPCR. Significance level for differences between groups was calculated by a *t*-test. **: *p* < 0.01. **a**. The gene *PSAP* and detected transcript CUFF.22759.5 in spliceR. **b**. *PSAP* transcript CUFF.22759.5 amplified product in agarose gel electrophoresis. **c**. RT-qPCR of *PSAP* transcript CUFF.22759.5 expression level. NC, non-challenged birds; C, challenged birds; M, marker

**Fig. 3:**
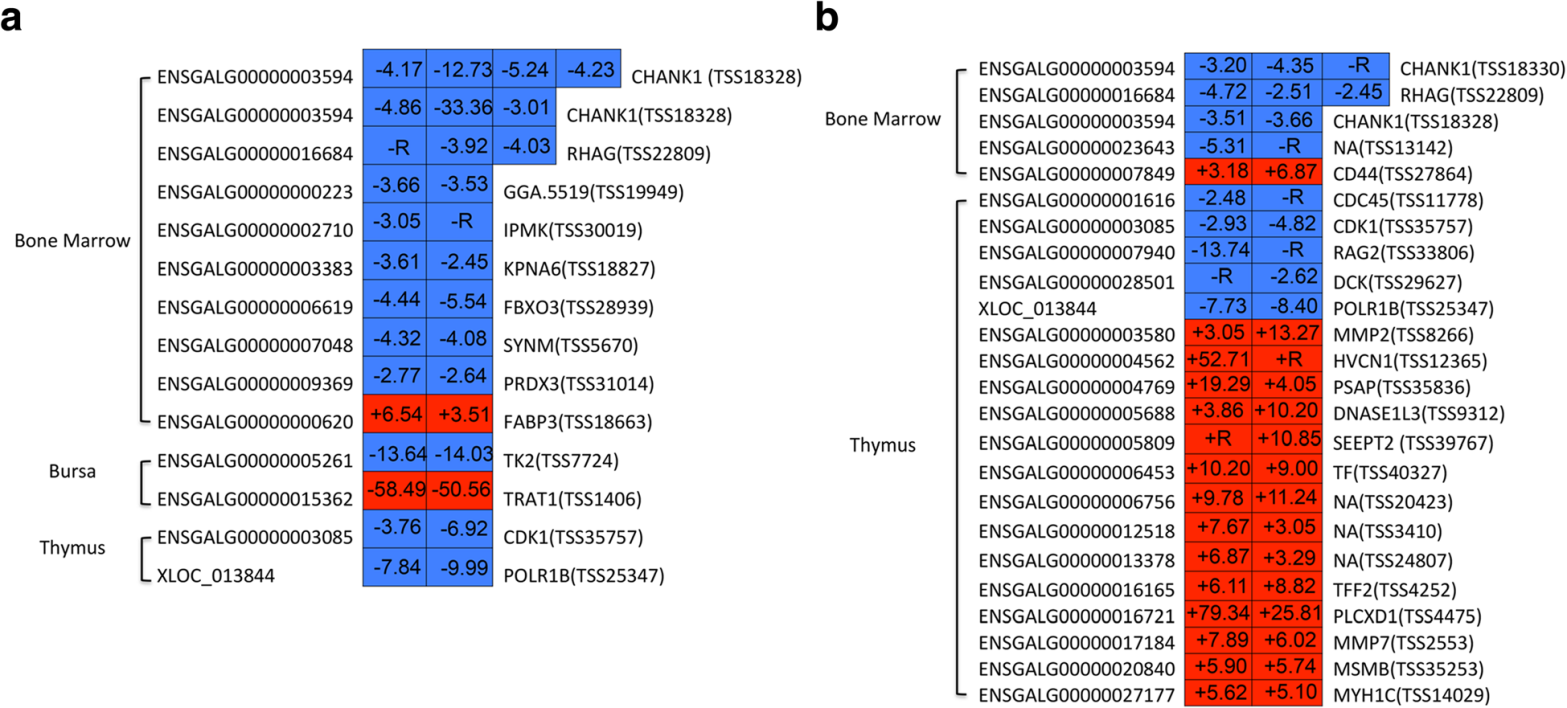
The isoforms containing gene from the same transcription start site in each of three primary lymphoid tissues in contrast susceptible vs. non-challenged birds at 5 days post-infection (dpi) and susceptible vs. resistant birds at 5 dpi. The *left* of heatmap is gene ID. The *right* of heatmap is gene name and transcription start site group id in parentheses. The number in *rectangle* of heatmap is the fold change of different isoforms. +, up-regulated; −, down-regulated; R, ∞. *Red color*, up-regulated isoforms. *Blue color*, down-regulated isoforms

The contrasting susceptible vs. non-challenged birds at 5 dpi had 174, 184, and 67 significant DE isoforms belonging to 145, 176, and 62 significant DE genes, generating 70, 56, and 22 novel isoforms as well as 9, 4, and 2 non-sense mediated decays (NMDs) in the bone marrow, bursa, and thymus, respectively (Additional file 1: Figure S1-3, Additional file 2: Table S1-3). For this contrast, 10, 2, and 12 DE genes from the same transcript start site (TSS) were found in the bone marrow, bursa, and thymus, respectively (Fig. 2a and Additional file 2: Table S4-6); 7, 6, and 2 DE genes from different TSSs were found in the bone marrow, bursa, and thymus (Fig. 3a and Additional file 2: Table S7-9).

When susceptible birds were contrasted with resistant birds at 5 dpi, 127, 24, and 610 significant DE isoforms belonging to 112, 23, and 577 significant DE genes, producing 46, 5, and 157 novel isoforms, as well as 2, 0, and 21 NMDs, were found in the bone marrow, bursa, and thymus, respectively (Additional file 1: Figure S3-5, Additional file 2: Table S10-12). In this contrast, 4 and 19 DE genes from the same TSS were found in the bone marrow and thymus, respectively (Fig. 2b and Additional file 2: Table S13-14). Meanwhile, 6, 1, and 13 DE genes came from different TSSs in the bone marrow, bursa, and thymus in the contrasting susceptible vs. resistant birds at 5 dpi (Fig. 3b and Additional file 2: Table S15-17).

The phagosome and lysosome pathways were the common pathways identified in susceptible birds that responded to systemic APEC infection in the bone marrow and thymus. Cell adhesion molecules (CAMs) were greatly induced in the bursa in contrasting susceptible vs. non-challenged birds at 5 dpi and in the thymus in the contrasting susceptible vs. resistant birds at 5 dpi (Table 1, Additional file 2: Table S18). The thymus in susceptible birds also expressed specific pathways to respond to systemic APEC infection: cytokine-cytokine receptor interaction, cell cycle, p53 signaling pathway, and apoptosis (Table 1, Additional file 2: Table S18). All of these detected significant pathways were consistent with the results obtained in previous single-tissue studies [15–17].

**Table 1:** Significantly changed pathways in each of the three primary lymphoid tissues in different contrasts

Contrast	Pathway	Adjusted *p* value		
		Bone Marrow	Thymus	Bursa
Susceptible vs. Non-challenged birds at 5 dpi	↑ gga 04145: Phagosome	1.34E-03	NA	NA
	↑ gga 04142: Lysosome	1.31E-02	NA	NA
	↓ gga 04110: Cell cycle	NA	6.98E-05	NA
	↓ gga 04115: p53 signaling pathway	NA	2.37E-02	NA
	↑ gga 04514: Cell adhesion molecules (CAMs)	NA	NA	5.48E-03
Susceptible vs. Resistant birds at 5 dpi	↑ gga 04145: Phagosome	2.29E-02	3.12E-02	NA
	↑ gga 04142: Lysosome	7.86E-03	1.18E-03	NA
	↑ gga 04060: Cytokine-cytokine receptor interaction	NA	2.58E-02	NA
	↑ gga 04210: Apoptosis	NA	2.14E-02	NA
	↓ gga 04110: Cell cycle	NA	7.30E-03	NA
	↑ gga 04514: Cell adhesion molecules (CAMs)	NA	3.26E-03	NA

*NA* none available, ↑ the significantly changed pathway was induced, ↓ the significantly changed pathway was suppressed

**Fig. 4:**
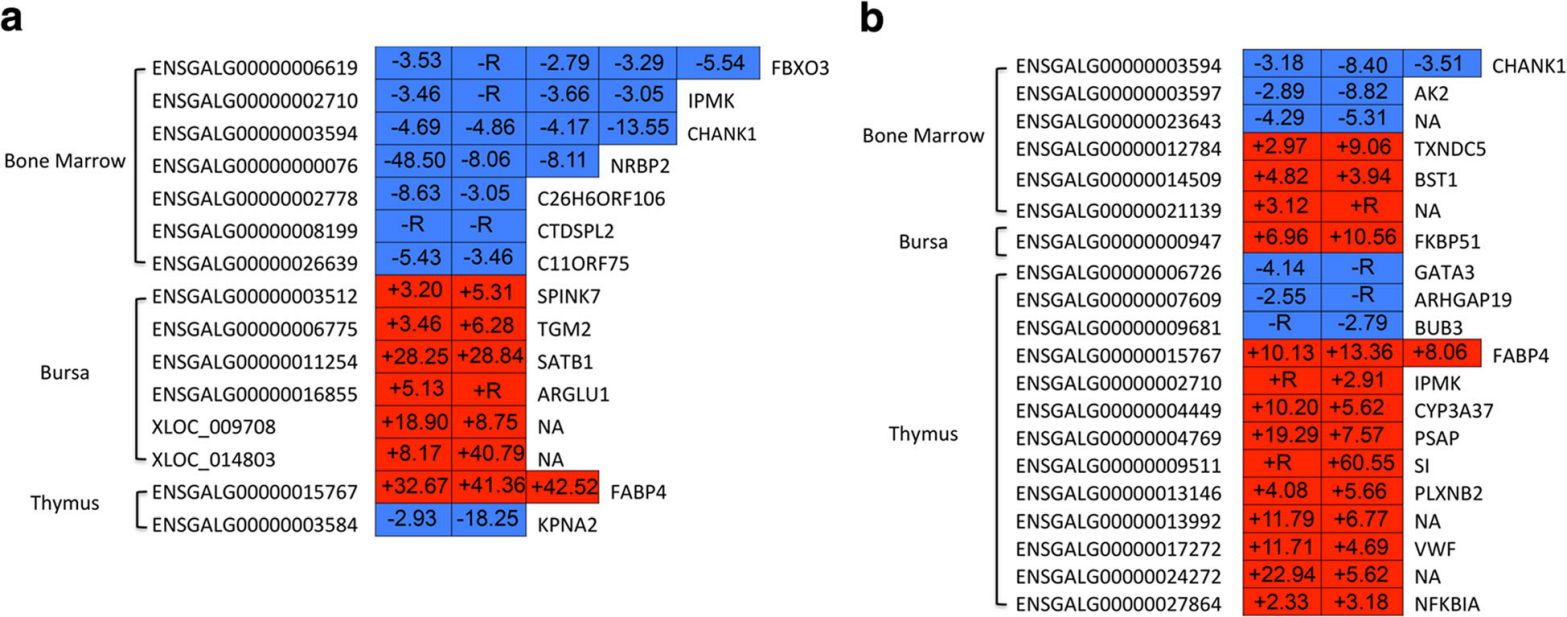
The isoforms containing gene from the different transcription start site in each of three primary lymphoid tissues in contrast susceptible vs. non-challenged birds at 5 days post-infection (dpi) and susceptible vs. resistant birds at 5 dpi. The *left* of heatmap is gene ID. The *right* of heatmap is gene name. The number in *rectangle* of heatmap is the fold change of different isoforms. +, up-regulated; −, down-regulated; R, ∞. *Red color*, up-regulated isoforms. *Blue color*, down-regulated isoforms

Two novel isoforms *PASP* (CUFF.22759.5) and *PASP* (CUFF.22759.2) involved in the lysosome pathway were detected in the present study. The novel isoform *PASP* (CUFF.22759.5) was also exhibited NMD. Validation of *PSAP* transcript CUFF.22759.5 was displayed in Fig. 2. Higher expression level of transcript CUFF.22759.5 was identified in challenged birds compared with that in non-challenged birds (Fig. 2b). In the cell cycle pathway, the isoform *STT3A* (ENSGALT00000001434) was found to exhibit NMD (Additional file 2: Table S18). Genes *CDK1* and *CDC45* participated in cell cycle and p53 signaling pathway were detected in several isoforms from the same TSS. The important isoforms (especially related to immune and growth response) from the same and different TSSs are presented in Figs. 3 and 4, such as *RAG2*, *POLR1B*, *DNASE1L3*, *TXND5*, and Ig lambda chain V-1 region.

## Discussion

This study provides a comprehensive description of the whole transcriptomic alternative splicing (AS) changes and non-sense mediated decay (NMD) in the primary lymphoid tissues of resistant and non-challenged birds compared with susceptible birds by using RNAseq data analysis. Thus, this study represents a powerful resource that enables further investigation into the molecular mechanisms of different host phenotypes in response to systemic APEC infection. AS events were generally ubiquitous in the transcriptome analysis of primary lymphoid tissues in chickens of different phenotypes. Exon skipping/inclusion (ESI) and alternative 5′ splice sites (A5SS) are predominant specific AS events in primary lymphoid tissues.

NMD is one of the conserved RNA-level post-transcriptional mechanisms for regulating gene expression pathway involved in development and stress response to exhibit RNA degradation and translational repression with premature termination [18, 19]. Both Rayson et al. [20] and Shi et al. [11] groups demonstrated the closer association of NMD with pathogen- or wounding-induced stresses. In the present study, two important genes (*PSAP* and *STT3A*) were involved in the significant pathways with NMD in the thymus of contrasting susceptible vs. resistant birds at 5 dpi. These two genes were significant DE genes and DE isoforms, referred to as differentially-expressed-transcript-containing genes (DETs).

In the current study on AS results, the *PSAP* gene gives rise to three different isoforms that have various structures involved in the lysosome pathway (Additional file 2: Table S18). *PSAP* is the precursor of lysosomal activator molecules as a cofactor to degradation of glycosphingolipids by lysosomal hydrolases [21–23]. Lysosomal *PSAP* breaks down proteins acting as biological catalysts and removes cells from internal and external waste. Mutation of the first half of the *PASP* C-terminus causes a problem in its transportation to lysosomes in the immune response [24]. *PSAP* had a crucial role in preventing cell death or apoptosis and promote cell survival [25, 26].

*PASP* also showed various responses in biological processes [27], such as the differentiation of male reproductive organs [27], spermatogenesis [28], and fertilization [29]. *PSAP* deficiency in human and mice can lead to death [30, 31]. Both Liu et al. [32] and Kramer et al. [33] reported the single nucleotide polymorphism (SNP) of *PSAP* was associated with spleen bacterial load under the *Salmonella* enteritidis challenge, indicating the *PSAP* is a positional candidate gene in response to pathogen-induced stress. In present study, two of the three *PSAP* isoforms were novel: CUFF.22759.2 (length = 4204 & FC = 7.56) and CUFF.22759.5 (length = 4143 & FC = 19.31). The isoform CUFF.22759.5 was found with NMD in the thymus of contrasting susceptible vs. resistant birds at 5 dpi (Additional file 2: Table S18).

Another DETs with NMD is *STT3A* involved in the significant cell cycle pathway in the thymus of contrasting susceptible vs. resistant birds at 5 dpi (Additional file 2: Table S18). This transcript ENSGALT00000001434 (*STT3A*) was significantly down-regulated (length = 1798 & FC = 3.36) in the thymus (Additional file 2: Table S18). *STT3A* plays an important role in DNA damage and replication checkpoints against spontaneous chromosome missegregation [34], which can protect the host against tunmorigenesis [35]. Modified *STT3A* was normally used for antimitotic chemotherapeutic drugs to kill tumor cells [35, 36]. Koiwa et al. [37] demonstrated that the *STT3A* subunit isoform has an important function control adaptive response to stress via reduction cell division in plant. On the basis of current results, DET *STT3A* was more highly expressed in the thymus of resistant birds than that in susceptible birds. Resistant birds could use the important gene *STT3A* to resist systemic APEC infection via NMD modification.

In addition to NMD, TSS is a crucial step to control gene expression activity under different conditions [38]. TSS was useful in understanding the transcription architecture and regulatory elements. Pal et al. [39] reported that the alternative TSS selection has a more important function in mRNA isoform diversity, compared with alternative splicing in some mammalian tissues because of TSS can turn on/off switches. Normally, the core promoters have a wide range of alternative TSS to start transcribing different isoforms [40–42].

Many interesting DETs with the same TSS were found in the thymus of contrasting susceptible vs. resistant birds at 5 dpi: *CDC45*, *CDK1*, *RAG2*, *POLR1B*, *PSAP*, and *DNASE1L3* (Fig. 2b and Additional file 2: Table S14). *CDC45* and *CDK1* were involved in the significant pathways cell cycle and p53 signaling pathway in this study (Additional file 2: Table S18). Recombination activating gene 2 (*RAG2*) has an important function in chicken B cells undergoing immunoglobulin gene conversion [43]. Polymerase (RNA) I polypeptide B (*POLR1B*) is a necessary enzyme for transcription. Deoxyribonuclease I-like 3 (*DNASE1L3*) is involved in immunoglobulin V gene diversification [44]. All aformentioned DETs were examined for down-regulation in the susceptible birds compared with the resistant birds at 5 dpi (Fig. 2b), indicating that DETs from cell death, cell cycle, cellular function, and maintenance were predominant under systemic APEC infection. Moreover, it is might be a key mechanism for resistant birds to resist APEC disease.

In the contrast of susceptible vs. resistant birds at 5 dpi, DETs, *TXNDC5* and the Ig lambda chain V-1 region (ENSGALG00000021139), were significantly up-regulated with different TSSs in the bone marrow (Fig. 3b and Additional file 2: Table S17). *TXNDC5* is involved in cell proliferation and migration [45] and is a potential pharmacological target [46]. In addition, the Ig lambda chain V-1 region contributes to the innate immune response [47]. In different tissues, alternative use of TSS is regulated in a specific pattern [48–50]. Under different conditions, altering the TSS choice can regulate protein synthesis to exhibit various biological effects [51]. Under systemic APEC infection, susceptible birds seem to have actively responded to the disease via properly expression of protein isoforms with different TSSs. In the present study, comprehensive detection of TSSs in different tissues can help elucidate the gene regulatory mechanisms and improve the prediction of gene regulation under systemic APEC infection.

## Conclusions

RNAseq and bioinformatics tools were used in this study to provide a portrait of the alternative splicing (AS) event and non-sense mediated decay (NMD) in primary lymphoid tissues, which can significantly influence host homeostasis under different conditions. This study indicates that AS plays a pivotal regulatory role in the immune response of chicken under systemic APEC infection via either NMD or alternative transcription start sites (TSSs). Two differentially-expressed-transcript-containing genes (DETs), *PSAP* and *STT3A*, were particularly important in this study because of their immune and growth response function. Many interesting DETs from the same and different TSS were detected, such as *CDC45*, *CDK1*, *RAG2*, *POLR1B*, *DNASE1L3*, *TXNDC5* and the Ig lambda chain V-1 region. Alternative TSSs seem to have an important function in pathogen-responsive expression patterns by regulating protein synthesis to exhibit various biological effects. The results of this study can be used to explore immune complexity and sheds light on the regulatory role of AS and NMD in systemic APEC infection, improving our understanding of the transcription architecture, genomic structure, and regulatory elements.

## Methods

### Experiment and RNAseq data

Briefly, one group of broilers was challenged with APEC O1 and the other group was non-challenged as control. Birds were euthanized with carbon dioxide gas followed by cervical dislocation at 1 day post-infection (dpi) and 5 dpi and bone marrow, thymus, and bursa were collected. At necropsy, a veterinary pathologist classified challenged birds as susceptible (total lesion score = 0–1) and resistant birds (total lesion score = 6–7) by using the standard pathology scoring system [52]: pericardium (0–2), liver (0–2), and air sacs (0–3). In total, six treatment groups were generated: susceptible birds at 1 and 5 dpi, resistant birds at 1 and 5 dpi, and non-challenged birds 1 and 5 dpi.

### RNAseq mapping reads and transcript assemble

Firstly, adapter for each read was removed via Fastx toolkit software v0.0.13. FastQC software v0.10.1 was used to evaluate reads quality for all samples with cutoff Phred score 32. NGS QC toolkit v2.2 was conducted to improve reads quality if the Phred score was lower than 32. After trimming adaptor and quality control, available reads of each sample were aligned to Ensemble gallus gallus 4.0 reference genome by using TopHat2 v2.0.9 with default parameters. TopHat2 is a popular and efficient tool to detect splice junction between exons [53, 54]. A local software of the Integrative Genomics Viewer v 2.2.17 [55] was performed to check the mapping results. The output of TopHat2 for each sample was in bam format, which was processed directly in the follow-up assembler program cufflinks v2.1.1 with default parameters to generate a transcriptome assembly. Cufflinks can use a known reference annotation to detect novel transcripts by assembling the reads into transcripts [56]. Fragments Per Kilobase of exon per Million fragments mapped (FPKM) was used to measured the transcript abundances through Cufflinks. After Cufflinks assemble, the assemblies of all samples for each tissue were merged together by using Cuffmerge v2.1.1 to provide a uniform basis for calculating gene and transcript expression. Moreover, the cuffcompare program was performed to test transcripts that are identical to the reference chicken genome (Ensembl gallus gallus 4.0 reference genome).

### Differential analysis and further biological pathway analysis

The Cuffmerge file along with alignment bam files produced by TopHat2 was used to conduct differential analysis between two treatment groups via Cuffdiff2 v2.1.1 with default parameters. Many simple tabular output files were generated by Cuffdiff2: FPKM tracking files (transcript FPKMs file, gene FPKMs file, primary transcript FPKMs file, and coding sequence FPKMs file), differential expression tests files (primary transcript differential FPKM file, transcript differential FPKM file, gene differential FPKM file, and coding sequence differential FPKM file), differential splicing tests file, differential coding output file, and differential promoter use file. CummeRbund v2.10.0 [57], a powerful R based visualization package, was used to create volcano, scatter, and box plots for the Cuffdiff2 output. In R package environment, CummeRbund can reorganize the output files of Cuffdiff2 to create a SQLite database about the relationships between genes, transcripts, transcription start sites (TSS), and coding sequences (CDS) regions. The cutoff for significant DE genes was q value < 0.05 and fold change (FC) was more than 1.5. Significant differentially expressed (DE) genes in Cuffdiff2 were used to identify the significantly changed pathways (adjusted *p* value < 0.05) by using GOseq.

### Alternative splicing detection

ASTALAVISTA v3.0 [58] was used to identify and classify the percent of the types of alternative splicing (AS) for each tissue using the merged assembled transcriptome gtf file. spliceR v1.10.0, a powerful R package freely available from the Bioconductor repository, was used to classify the alternative splicing and predict the coding potential from RNAseq data [59]. This package can detect single and multiple exon skipping/inclusion (ESI and MESI), intron retention/inclusion (ISI), alternative 5′/3′ splice sites (A5SS/A3SS), alternative transcription start site (ATSS), transcription termination site (ATTS), mutually exclusive exons (MEE), as well as the nonsense mediated decay (NMD) sensitivity of transcripts based on stop codon position. Also, this software can detect the different isoforms within a gene whether were from the same TSS or different TSS. Visualization and the generation of GTF files for genome browsers can be also obtained from spliceR. Moreover, the significant DE isoform was q value < 0.05 and fold change > 1.5.

### Validation of alternative splicing by RT-qPCR

To validate the alternative splicing (AS) results, independent samples (challenged and non-challenged birds) were used to detect the relative expression level of transcript CUFF.22759.5 (*PSAP*) in thymus. After isolating the total RNA, RT-qPCR was used to amplify the splicing isoform by using one primer in the alternative exon and an opposing primer in a constitutive exon (Fig. 2a). Moreover, we used a boundary-spanning primer for the sequence encompassing the exon–exon junction with the opposing primer in a constitutive exon in this validated study.

Total RNA was extracted from different phenotype birds using TRIZOL Reagent according to manufacturer’s instructions (Life Technologies, CA, US). Isolated RNA was treated with RNase-free Dnase I to remove DNA contamination. A nanodrop spectrophotometer was used to measure the quantity and quality of purified RNA. The primers sequence of *PSAP* (CUFF.22759.5) was: F, 5- GGCTGTTGACCCTGCTT-3; R, 5-GATATGGCTGTGTTCAATAATGCA-3. QuantiTect SYBR Green RT-PCR kit (Qiagen Inc., Valencia, CA) was used to test transcript expression. 28S rRNA, a housekeeping gene, was served as internal control. Gene assay was run in triplicate for the same individual samples in RT-qPCR. The reaction products were resolved on 2% TAE/agarose gels. All of the candidate fragments were cloned for sequencing to check transcript CUFF.22759.5. The adjusted cycle threshold (Ct) values were calculated using the equation: 40 – [Ct sample gene mean + (Ct 28S median – Ct 28S mean) (slope of sample gene/slope of 28S)]. The qPCR data were analyzed using JMP 8.0.2 statistical software (SAS Institute Inc., Cary, NC).

## Additional files

Additional file 1:
**Figure S1.** The significant differentially expressed (DE) isoforms and DE genes in the nine contrast of bone marrow. S5, susceptible birds at 5 days post-infection; S1, susceptible birds at 1 day post-infection; R5, resistant birds at 5 days post-infection; R1, resistant birds at 1 day post-infection; NC5, non-challenged birds at 5 days post-infection; NC1, non-challenged birds at 1 day post-infection. Pre-mature stop codon (PTC) is equal to non-sense mediated decay (NMD). **Figure S2.** The significant differentially expressed (DE) isoforms and DE genes in the nine contrast of bursa. S5, susceptible birds at 5 days post-infection; S1, susceptible birds at 1 day post-infection; R5, resistant birds at 5 days post-infection; R1, resistant birds at 1 day post-infection; NC5, non-challenged birds at 5 days post-infection; NC1, non-challenged birds at 1 day post-infection. Pre-mature stop codon (PTC) is equal to non-sense mediated decay (NMD). **Figure S3.** The significant differentially expressed (DE) isoforms and DE genes in the nine contrast of thymus. S5, susceptible birds at 5 days post-infection; S1, susceptible birds at 1 day post-infection; R5, resistant birds at 5 days post-infection; R1, resistant birds at 1 day post-infection; NC5, non-challenged birds at 5 days post-infection; NC1, non-challenged birds at 1 day post-infection. Pre-mature stop codon (PTC) is equal to non-sense mediated decay (NMD). **Figure S4.** Raw, clean, unique mapped, and splice reads distribution in each treatment in each of the three immune tissues. NC1, non-challenged birds at 1 day post-infection; NC5, non-challenged birds at 5 days post-infection; R1, resistant birds at 1 day post-infection; R5, resistant birds at 5 days post-infection; S1, susceptible birds at 1 day post-infection; S5, susceptible birds at 5 days post-infection (DOCX 2498 kb)
Additional file 2:
**Table S1.** Significant DEI and DEG in susceptible vs. non-challenged birds at 5 dpi in bone marrow. **Table S2.** Significant DEI and DEG in susceptible vs. non-challenged birds at 5 dpi in bursa. **Table S3.** Significant DEI and DEG in susceptible vs. non-challenged birds at 5 dpi in thymus. **Table S4.** DEG from the same TSS in susceptible vs. non-challenged birds at 5 dpi in bone marrow. **Table S5.** DEG from the same TSS in susceptible vs. non-challenged birds at 5 dpi in bursa. **Table S6.** DEG from the same TSS in susceptible vs. non-challenged birds at 5 dpi in thymus. **Table S7.** DEG from different TSS in susceptible vs. non-challenged birds at 5 dpi in bone marrow. **Table S8.** DEG from different TSS in susceptible vs. non-challenged birds at 5 dpi in bursa. **Table S9.** DEG from different TSS in susceptible vs. non-challenged birds at 5 dpi in thymus. **Table S10.** Significant DEI and DEG in susceptible vs. resistant birds at 5 dpi in bone marrow. **Table S11.** Significant DEI and DEG in susceptible vs. resistant birds at 5 dpi in bursa. **Table S12.** Significant DEI and DEG in susceptible vs. resistant birds at 5 dpi in thymus. **Table S13.** DEG from the same TSS in susceptible vs. resistant birds at 5 dpi in bone marrow. **Table S14.** DEG from the same TSS in susceptible vs. resistant birds at 5 dpi in thymus. **Table S15.** DEG from different TSS in susceptible vs. resistant birds at 5 dpi in bone marrow. **Table S16.** DEG from different TSS in susceptible vs. resistant birds at 5 dpi in bursa. **Table S17.** DEG from different TSS in susceptible vs. resistant birds at 5 dpi in thymus. **Table S18.** Significant DEI in the significantly changed pathways in different contrasts in different tissues. (XLSX 904 kb)

## Abbreviations

A3SS: Alternative 3′ splice sites
A5SS: Alternative 5′ splice sites
APEC: Avian pathogenic *Escherichia coli*
AS: Alternative splicing
ATSS: Alternative transcription start site
ATTS: Transcription termination site
CAM: Cell adhesion molecules
CDS: Coding sequences
DE: Differentially expressed
DET: Differentially-expressed-transcript-containing gene
ESI: Exon skipping/inclusion
ExPEC: Extraintestinal pathogenic *Escherichia coli*
FC: Fold change
FPKM: Fragments Per Kilobase of exon per Million fragments mapped
GEO: Gene expression omnibus
IR: Intron retention
MEE: Mutually exclusive exons
NMD: Nonsense-mediated decay
TSS: Transcription start site
