# Supplementary material for: Deciphering alternative splicing and nonsense-mediated decay modulate expression in primary lymphoid tissues of birds infected with avian pathogenic E. coli (APEC)
Source: BMC Genet. 2017 Mar 7;18:21. doi: 10.1186/s12863-017-0488-4 (PMC5341183; doi:10.1186/s12863-017-0488-4)
Supplement: Additional file 1: — Figure S1. The significant differentially expressed (DE) isoforms and DE genes in the nine contrast of bone marrow. S5, susceptible birds at 5 days post-infection; S1, susceptible birds at 1 day post-infection; R5, resistant birds at 5 days post-infection; R1, resistant birds at 1 day post-infection; NC5, non-challenged birds at 5 days post-infection; NC1, non-challenged birds at 1 day post-infection. Pre-mature stop codon (PTC) is equal to non-sense mediated decay (NMD). Figure S2. The significant differentially expressed (DE) isoforms and DE genes in the nine contrast of bursa. S5, susceptible birds at 5 days post-infection; S1, susceptible birds at 1 day post-infection; R5, resistant birds at 5 days post-infection; R1, resistant birds at 1 day post-infection; NC5, non-challenged birds at 5 days post-infection; NC1, non-challenged birds at 1 day post-infection. Pre-mature stop codon (PTC) is equal to non-sense mediated decay (NMD). Figure S3. The significant differentially expressed (DE) isoforms and DE genes in the nine contrast of thymus. S5, susceptible birds at 5 days post-infection; S1, susceptible birds at 1 day post-infection; R5, resistant birds at 5 days post-infection; R1, resistant birds at 1 day post-infection; NC5, non-challenged birds at 5 days post-infection; NC1, non-challenged birds at 1 day post-infection. Pre-mature stop codon (PTC) is equal to non-sense mediated decay (NMD). Figure S4. Raw, clean, unique mapped, and splice reads distribution in each treatment in each of the three immune tissues. NC1, non-challenged birds at 1 day post-infection; NC5, non-challenged birds at 5 days post-infection; R1, resistant birds at 1 day post-infection; R5, resistant birds at 5 days post-infection; S1, susceptible birds at 1 day post-infection; S5, susceptible birds at 5 days post-infection (DOCX 2498 kb) [file 12863_2017_488_MOESM1_ESM.docx]

Figure 1. The significant differentially expressed (DE) isoforms and DE genes in the nine contrast of bone marrow. S5, susceptible birds at 5 days post-infection; S1, susceptible birds at 1 day post-infection; R5, resistant birds at 5 days post-infection; R1, resistant birds at 1 day post-infection; NC5, non-challenged birds at 5 days post-infection; NC1, non-challenged birds at 1 day post-infection. Pre-mature stop codon (PTC) is equal to non-sense mediated decay (NMD).

Figure 2. The significant differentially expressed (DE) isoforms and DE genes in the nine contrast of bursa. S5, susceptible birds at 5 days post-infection; S1, susceptible birds at 1 day post-infection; R5, resistant birds at 5 days post-infection; R1, resistant birds at 1 day post-infection; NC5, non-challenged birds at 5 days post-infection; NC1, non-challenged birds at 1 day post-infection. Pre-mature stop codon (PTC) is equal to non-sense mediated decay (NMD).

Figure 3. The significant differentially expressed (DE) isoforms and DE genes in the nine contrast of thymus. S5, susceptible birds at 5 days post-infection; S1, susceptible birds at 1 day post-infection; R5, resistant birds at 5 days post-infection; R1, resistant birds at 1 day post-infection; NC5, non-challenged birds at 5 days post-infection; NC1, non-challenged birds at 1 day post-infection. Pre-mature stop codon (PTC) is equal to non-sense mediated decay (NMD).

Fig. 4 Raw, clean, unique mapped, and splice reads distribution in each treatment in each of the three immune tissues. NC1, non-challenged birds at 1 day post-infection; NC5, non-challenged birds at 5 days post-infection; R1, resistant birds at 1 day post-infection; R5, resistant birds at 5 days post-infection; S1, susceptible birds at 1 day post-infection; S5, susceptible birds at 5 days post-infection.
